# An Empirical Study on Transmission Beamforming for Ultrasonic Guided-Wave Based Structural Health Monitoring

**DOI:** 10.3390/s20051445

**Published:** 2020-03-06

**Authors:** Sergio Cantero-Chinchilla, Gerardo Aranguren, Muhammad Khalid Malik, Josu Etxaniz, Federico Martín de la Escalera

**Affiliations:** 1 Institute for Aerospace Technology & The Composites Group, The University of Nottingham, Nottingham NG7 2RD, UKMuhammad.Malik@nottingham.ac.uk (M.K.M.); 2 Aernnova Engineering Division S.A., 28034 Madrid, Spain; federico.martindelaescalera@aernnova.com; 3 Electronic Design Group, University of the Basque Country (UPV/EHU), 48013 Bilbao, Spain; josu.etxaniz@ehu.es

**Keywords:** SHM, structural inspection, ultrasonic guided-waves, transmission beamforming, phased-array

## Abstract

The development of reliable structural health monitoring techniques is enabling a healthy transition from preventive to condition-based maintenance, hence leading to safer and more efficient operation of different industries. Ultrasonic guided-wave based beamforming is one of the most promising techniques, which supports the monitoring of large thin-walled structures. However, beamforming has been typically applied to the post-processing stage (also known as virtual or receiver beamforming) because transmission or physical beamforming requires complex hardware configurations. This paper introduces an electronic structural health monitoring system that carries out transmission beamforming experiments by simultaneously emitting and receiving ultrasonic guided-waves using several transducers. An empirical characterization of the transmission beamforming technique for monitoring an aluminum plate is provided in this work. The high signal-to-noise ratio and accurate angular precision of the physical signal obtained in the experiments suggest that transmission beamforming can increase the reliability and robustnessof this monitoring technique for large structures and in real-world noisy environments.

## 1. Introduction

Industry requires the testing of materials to analyze their properties and health condition. Some of the tests are destructive and often aimed at determining their physical properties, such as fracture toughness and fatigue strength. However, when the specimens are tested to comply with certain standards but they still need to be used in later operation, non-destructive testing (NDT) methods are required [1], such as Liquid Penetrant Testing, Ultrasonic Testing, and Visual Testing [2]. These techniques are typically used during manufacturing processes and in-service inspections. The latter can be framed within scheduled maintenance actions, which usually imply a high cost for the operator.

Alternatively, condition-based maintenance using structural health monitoring (SHM) data is becoming more popular among different industries due to their potential economical and safety-related benefits [3]. Among the available SHM techniques, such as ultrasonic guided-waves, acoustic emission, and vibration analysis, the first one stands out due to their ability to explore large areas with relatively low attenuation [4]. Note that vibration-based methods (e.g., first-order eigen-perturbation and Kalman filter techniques [5]) provide efficient solutions for real-time SHM of very large structures, such as bridges or buildings [6]. However, ultrasonic guided-wave based techniques are generally more appropriate for the early stage detection of small damages [7]. This is particularly relevant in the aerospace industry, where ultrasonic guided-waves are typically applied to monitor safety-critical thin-walled structures such as metallic or composite panels [8]. It is worth mentioning that the main objective of these techniques is to detect, localize, and identify any kind of hazardous inhomogeneity in the structure by analyzing the measured data with appropriate post-processing methods [9], such as artificial neural networks [4] and probabilistic Bayesian approaches [10].

Ultrasonic guided-waves are generated by converting electrical signals into mechanical waves using piezoelectric wafer active sensor (PWAS). These sensors are able to transduce mechanical displacements into electrical signals, and hence they can be used as emitters and receivers. The SHM using ultrasonic guided-waves is typically addressed by permanently attaching several PWAS to a plate-like structure, while a SHM ultrasonic system (SHMUS) manages the generation, acquisition, and post-processing of the ultrasonic signals. This technique may be performed using a pitch–catch approach, e.g., using two PWAS, one as emitter and the other as receptor, or a pulse-echo approach, i.e., relying both generation and acquisition on the same PWAS. The use of one emitter PWAS for SHM entails significant limitations. The energy of the transmitted signal using only one PWAS is relatively small. Besides, the wave is attenuated depending on the propagated distance. Therefore, the received signals typically have small amplitudes and low signal-to-noise ratio (SNR). These limitations can be generally overcome by using arrays of PWAS, which are able to supply higher energy and, therefore, more accurate level of information for large structures [9].

These arrays of PWAS can be used by testing methods that only utilize one emitter PWAS for each test, while the rest act as receivers, such as the round-robin method [11]. In this context, signal post-processing approaches are needed to sum up the signals for different times of flight, thus creating a virtual representation of the beamforming technique, also known as receiver beamforming [12]. Alternatively, the only technique that can use multiple PWAS transducers simultaneously as emitters is the transmission beamforming [12], which is particularly difficult to implement in practice. The transmission beamforming technique allows to obtain a comparatively greater amplitude of the wave by using a whole array of PWAS simultaneously in generation [9,11]. It is able to create a beam from the constructive interference of different wave packets by introducing a phase difference between the ultrasonic signals. By modifying such phase, the main beam can be steered in different directions to sweep a large area [13]. Transmission beamforming has been scarcely used in practice, while receiver beamforming has been widely studied in the literature due to the ease of experimental implementation. Unfortunately, receiver beamforming still has all the limitations of round-robin method due to the use of only one emitter at a time. In contrast, transmission beamforming is able to overcome these issues by generating greater amplitudes than the ones created by receiver beamforming, and consequently obtaining higher SNR. Given the lack of practical implementations that allow the use of this promising technique, an empirical characterization of transmission beamforming in plate-like structures for their continuous SHM is still missing in the literature.

**Figure 1:**
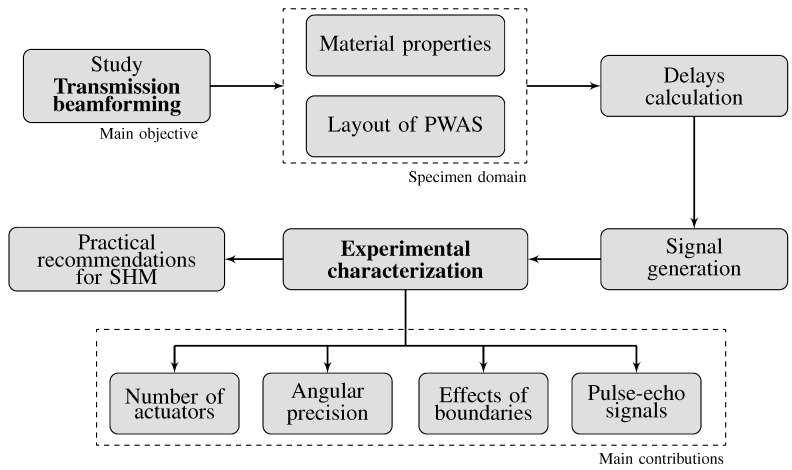
Main objective, contributions, and methodology proposed in this paper.

The main objective of this paper is to illustrate the advantages of the transmission beamforming technique applied to ultrasonic guided-waves in an experimental setup for isotropic materials. Additionally, the main contribution lies in the experimental characterization and proof-of-concept of the transmission beamforming technique (see Figure 1) using a relatively high number of actuators (up to ten) in a linear phased-array. This is achieved using a dedicated SHMUS hardware for both the generation and acquisition of ultrasonic signals in phase. This system relies on an integrated solution for the management of several channels able to emit and receive ultrasonic signals simultaneously. A linear phased-array of ten PWAS is permanently attached to an aluminum plate. They can be used to generate phased signals to focus the beam at a point of interest or in a specific direction. Additional PWAS are placed along the structure so that the performance of the transmission beamforming technique can be evaluated. 

The experimental characterization proposed here consists of studying the following attributes of transmission beamforming in isotropic materials: (i) the effect of the number of PWAS in the phased-array on the amplitude of the constructive interference, (ii) the width and attenuation of the main beam, (iii) the consequences of the boundary reflections on the accuracy of transmission beamforming near the edge of the structure, and (iv) the amplitude of the wave packets acquired in pulse-echo mode. In general, greater amplitudes are obtained at the point of interest using a fast and practical hardware, which potentially entails efficient and reliable SHM of large structures in real-world engineering scenarios.

The remainder of the manuscript is outlined as follows. The background and fundamentals of the ultrasonic guided-wave based beamforming technique are described in Section 2. Next, the experimental setup including the description of the SHMUS and the aluminum plate are presented in Section 3. Then, the experimental results along with their practical implications are assessed in Section 4. Finally, Section 5 provides concluding remarks and practical recommendations for SHM.

## 2. Phased-Array Systems for Beamforming

### 2.1. Background of Beamforming Techniques

The receiver beamforming technique aligns the acquired signals in several round-robin tests by introducing a delay. This delay is calculated using the time of flight from each PWAS in an array to a specific position of the plate [14]. Then, these signals are added using the well-known Delay-and-Sum (DAS) algorithm [15]. This creates higher synthetic amplitudes whereby a potential defect can be more easily identified. This technique has been widely studied in the literature due to the ease of implementation. The experimental setup requires only one actuator and a number of sensors in an array to perform the virtual beamforming [16]. For instance, the performance of 1D and 2D geometries of arrays of PWAS transducers has been comparatively studied in order to obtain the ones with lower side-lobe effects [17]. The experiments were carried out using a data acquisition system, a signal generator and a multiplexor unit that controlled PWAS transducers in order to perform round-robin tests. The ultrasonic signals were then used for the imaging algorithm. Other geometries of PWAS arrays have also been explored. This includes circular arrays with a single actuator in the center [18] or using uniform and circular arrays in passive mode for localizing acoustic emission sources, i.e., using the PWAS transducers in listening mode [19]. Additionally, the performance analysis of receiver beamforming has been carried out in composite structures. A laser vibrometer is used for acquiring ultrasonic signals. The specific guided-wave parameters and the energy skew effects are also taken into account for anisotropic materials [20]. The use of receiver bemforming and laser Doppler vibrometer have been further explored in damage detection and quantification in composite structures [21], and in an improved damage identification approach in aluminum plates by a modified DAS algorithm [22].

Adaptive methods arise to enhance the image reconstruction and reduce side-lobe effects by applying weighting coefficients to the signals received in the array PWAS transducers [23,24]. Although they may be applied for both transmission and receiver beamforming, its use in the latter has been studied more in the literature. For instance, the adaptive technique has been used for the development of an improved imaging algorithm using distributed arrays of PWAS and a multipath approach in both aluminum and composite plates, without the need of material information [25,26]. Furthermore, this beamforming mode has been applied to laser vibrometer measurements along with a baseline-free imaging algorithm [27]. Alternatively, the adaptive method has been recently applied for transmission beamforming, whereby a recursive approach has been used for the generation of the main beam in two directions [28]. However, most of the receiver beamforming based approaches show a limitation for real-world SHM applications, which typically require a light-weight and integrated device. Such a device should be able to both excite and acquire PWAS transducers with a relatively low post-processing burden and have the ability to work in noisy environment.

To provide comparatively higher amplitude in the acquired signals, which is suitable for noisy working environments, the transmission beamforming technique may be used. However, it has typically required a more complex signal generation system [29,30,31,32]. Thus, its use has been restricted to a limited number of contributions, which have focused on the development of the proof-of-concept with conventional equipment (e.g., using waveform generator cards) [12]. More recently, compact phased-arrays in triangular layouts have been used, whereby two PWAS transducers are used as actuators, and a third one as sensor [29]. Additionally, compared to receiver beamforming, transmission beamforming has the advantage to generate different geometries of beams, such as the bottle shaped beam and the vortex beam, which may be helpful for SHM [33]. Still, there is an evident need for a dedicated hardware that is able to perform transmission beamforming using a higher number of PWAS actuators for an enhanced SHM.

### 2.2. Transmission Beamforming Fundamentals

The transmission beamforming technique is carried out by emitting multiple synchronized signals with a calculated phase difference in terms of time delay. Such signals then go to the array of PWAS transducers, which cause the guided-waves to propagate and sum up at either an arbitrary point or a certain direction in a thin-walled structure. The structure can then be entirely inspected by sending a beam at several directions, by modifying the time delays. Finally, a structural health condition assessment obtained by the post-processing of acquired ultrasonic signals can be provided. The use of DAS algorithm is not necessary at the post-processing stage as the signals have summed up physically during the excitation phase.

**Figure 2:**
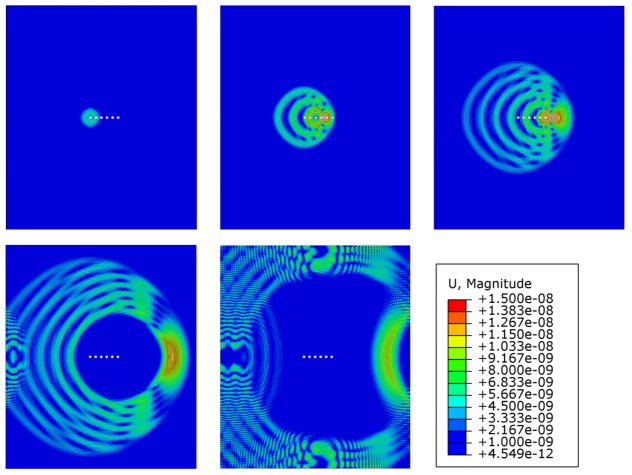
Transmission beamforming simulation using a linear phased-array of 6 piezoelectric wafer active sensors (PWASs) (white circles) focusing horizontally towards right of the plate.

The time delays for isotropic materials are calculated considering the following aspects: (i) the mechanical properties of the plate-like structure, which dictates the wave propagation velocity of the guided-wave mode of interest, (ii) the relative position of the PWAS transducers in the array, and (iii) if transmission beamforming is performed to focus on one specific point of the structure or if it is steered towards a direction and thus intersecting in the infinite [34]. Considering a structure made of isotropic material, the delay $\delta_{i}$ for the signal generated at the *i*-th transducer in the array for transmission beamforming in a direction $\vec{\xi }$ is given by [35]:

(1) $$\begin{array}{c} \delta_{i}=-\frac{{\vec{s}}_{i}\;\cdot \;\vec{\xi }}{V(f)} \end{array}$$

where ${\vec{s}}_{i}$ is the vector from the origin of coordinates, which is normally assumed to be at the center of the array, to the *i*-th transducer of the array. $\vec{\xi }$ is the vector that defines the direction of the beam and V(f) is the wave propagation velocity at a frequency *f*. Figure 2 illustrates a transmission beamforming simulation performed with a linear phased-array of 6 PWAS transducers steering the beam towards the horizontal direction in an aluminum plate. The simulation is performed in Abaqus using the explicit dynamics solver. The simulation setup is given in detail in Appendix A. The main beam of the first antisymmetric mode is focused towards the right by introducing a delay between the excitation signals. On the other hand, it is possible to inspect specific points using the delays calculated as follows [35]:

(2) $$\begin{array}{c} \delta_{i}=-\frac{d_{0k}-d_{ik}}{V(f)} \end{array}$$

where $d_{0k}$ is the distance between the origin of coordinates and the *k*-th point of interest, and $d_{ik}$ is the distance between the *i*-th element of the phased-array and the point of interest. In this work, the beamforming approach to focus on a direction $\vec{\xi }$ is used due to its generic character for monitoring a plate-like structure of different dimensions. Note that the use of this approach allows to sweep an area without prior knowledge of its geometry, while focusing at specific points would require to establish a grid of monitoring points which are case specific. The wave propagation velocity is obtained through a preliminary experiment whereby one PWAS is excited and two additional aligned sensors are used to acquire the ultrasonic signal. Once the signals are measured, the time of flight between consecutive sensors is used to calculate the wave propagation velocity of the principal guided-wave mode at the frequency of excitation. These measurements are repeated several times so that an averaged and more robust estimate of the velocity is obtained.

## 3. Experimental Setup

The experimental setup consists of two main parts, a SHMUS able to generate, acquire and store electrical signals and a structure that is interrogated by the hardware part. The latter also includes the PWAS transducers that convert such electrical signals into mechanical displacements and vice versa.

### 3.1. Hardware of the SHMUS

The execution of ultrasonic guided-wave based tests for SHM requires an electronic system, which can be built from conventional or dedicated instrumentation. Conventional instrumentation typically consists of a number of independent equipment such as an arbitrary signal generator, a data acquisition system, and a signal amplifier. However, factors such as their size or weight make them only applicable to laboratory-based activities, hence limiting their use in real-world engineering scenarios, where the equipment needs to be efficient and portable. In addition, the delayed excitation of the actuators using such conventional equipment is limited with respect to the time precision of the necessary synchronization. To overcome such drawbacks, a dedicated hardware for generation and acquisition of ultrasonic signals for the purpose of SHM is used here [36].

**Figure 3:**
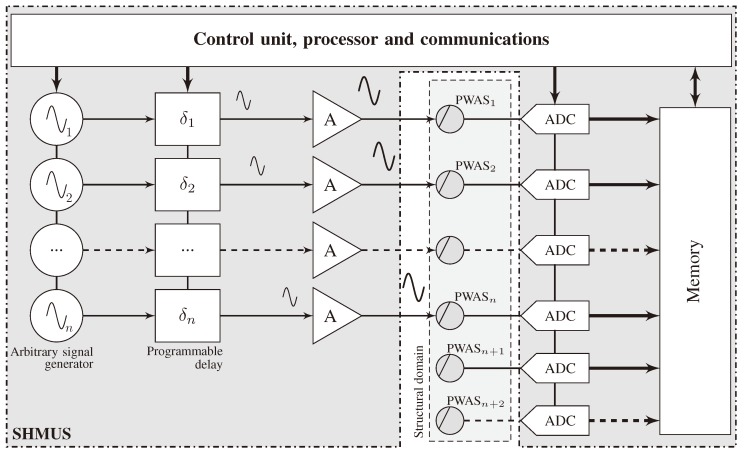
Generic scheme of the hardware needed to carry out transmission beamforming. PWAS 1,2,…,n are used in pulse-echo mode. Besides, PWAS n+1,n+2,… are operated in pitch–catch mode.

The SHMUS is depicted in Figure 3 along with the sensors and the structure. A control unit, which contains the processor and predefined internal communications, is used to command the rest of the elements in the experimental setup. Such control unit internally generates *n* electrical signals using the internal arbitrary signal generators, which are then adjusted by introducing previously calculated delays $\left(\delta_{1},\delta_{2},\ldots ,\delta_{n}\right)$. Note that each channel contains an independent signal generator. The delays are calculated so that constructive interferences of the guided-waves are used to examine a plate-like structure in a similar way as a radar works [37], as shown hereinafter. The delayed signals are then amplified before reaching the PWAS transducers that are attached to the plate-like structure. Thus, the electrical signals are transformed into mechanical displacements of the structure that propagates according to both the plate’s mechanical characteristics and the frequency of excitation.

At the same time that the PWAS transducers excite the structure, they start acquiring the mechanical displacements. Such response may vary depending on the test mode, which may typically be pulse-echo (e.g., PWAS${}_{1}$ to PWAS${}_{n}$ in Figure 3) or pitch–catch (e.g., PWAS${}_{n+1}$ and PWAS${}_{n+2}$). The acquired mechanical displacements are transformed into electrical signals by the PWAS transducers. Such signals are then digitized by means of analog-digital converters, and stored in the memory, which are managed by the control unit.

In the experiments, phased-array monitoring for enhanced life assessment (PAMELA) version 4 has been used as SHMUS. It has 18 independent channels available for generation and acquisition, which can be simultaneously operated. Sinusoidal signals can be generated from 30 kHz to 3 MHz frequency, up to 48 V${}_{\mathrm{pp}}$ of amplitude, and up to 1 W of power per channel during the excitation. The input channels have 1 V${}_{\mathrm{pp}}$ of full scale and they are sampled at 60 MSPS with 12 bits of resolution.

### 3.2. Setup of the Isotropic Plate-Like Structure

**Figure 4:**
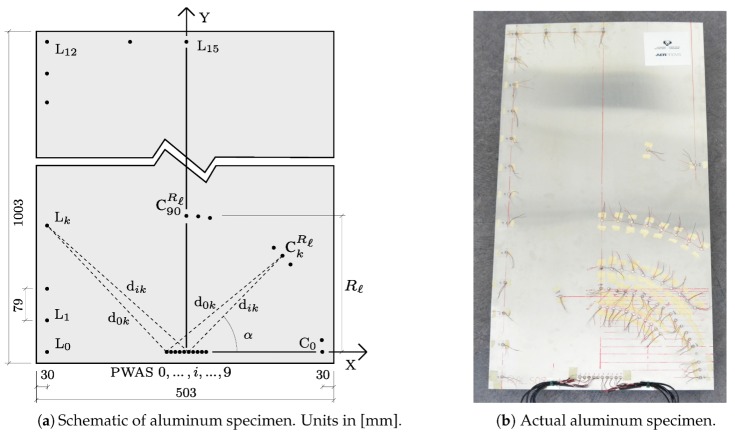
Aluminum specimen for transmission beamforming tests.

The plate-like structure used for assessing the performance of the transmission beamforming technique is a QQ-A-250/5 ‘O’ aluminum plate, a medium to high strength alloy with Young’s modulus E=73GPa, density $\rho =2740\;{\mathrm{kg}/m}^{3}$, and Poisson’s ratio ν=0.33. This isotropic material entails the wave propagation velocity to be constant in all directions, thus making the time delay calculation analogous at every steering direction. Figure 4a schematically depicts the aluminum plate of 1mm thickness along with the position of the PWAS transducers that were placed in the structure. Figure 4b shows the real specimen, the phased-array and the set of PWAS used in the experiments. Note also that the PWAS transducers need to be carefully selected in order to generate guided-waves in a structure. In general, the main factors involved are the frequency of excitation at which the test is to be carried out and the predominant guided-wave mode that is desired to interrogate the structure. The first symmetric mode is selected in this study given its higher amplitude when generated by the PWAS transducers. Here, PWAS discs of 7mm diameter and 0.2mm thickness with radial mode vibration and a resonant frequency at 300kHz supplied by STEMINC (part number SMD07T02R412WL) are used to both generate and acquire the ultrasonic guided-waves.

The array of ten PWAS transducers controlled by the SHMUS was symmetrically centered at the bottom of the plate (separated 30mm from the edge). This position emulates a typical industrial imposition, whereby the system needs to be close to the edge of the monitored structure for accessibility reasons (e.g., to carry out a maintenance action). The elements of the array are numbered by (0,1,…,i,…,9) starting from the left-hand side, as shown in Figure 4a. A series of PWAS sensors were distributed all over the plate in order to assess the transmission beamforming technique in different areas of the structure. The sensors denoted as $\left\{C_{0}^{R_{\ell }},C_{5}^{R_{\ell }},\ldots ,C_{k}^{R_{\ell }},\ldots ,C_{90}^{R_{\ell }}\right\}$ are evenly spaced every $5^{{}^{\circ}}$ and placed over three circumferential paths (with origin at the center of the phased-array) with radius $R_{1}=170\;\mathrm{mm}$, $R_{2}=270\;\mathrm{mm}$, and $R_{3}=400\;\mathrm{mm}$, respectively. The maximum amplitude of the main lobe was then measured in all of these sensors when steering the main beam at different directions. Besides, another set of sensors were placed following a Cartesian layout at both left and top plate edges, namely $\left\{L_{0},L_{1},\ldots ,L_{k},\ldots ,L_{15}\right\}$, starting at the bottom left corner ($L_{0}$) up to the top center of the plate ($L_{15}$). These evenly spaced sensors every 79mm are used to investigate the suitability of the beamforming technique in interrogating the edges of the plate.

**Figure 5:**
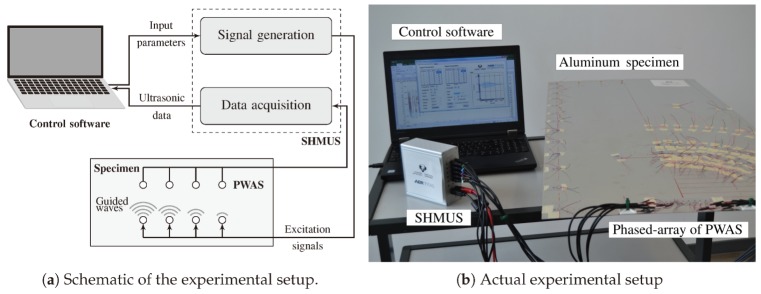
Experimental setup used in the transmission beamforming tests.

The experimental setup used in these tests is shown in Figure 5. The phased-array of PWAS transducers is connected to the SHMUS by a shielded twisted-pair cable, which is managed by a control software installed in a laptop and connected via USB 2.0. The PWAS sensors placed along the rest of the structure for the assessment of the beamforming technique are also connected to the SHMUS so that the data of interest are acquired when required.

### 3.3. Generation of Ultrasonic Signals

Once the wave propagation velocity of the symmetric 0 (S0) mode obtained, with a value of 5492.26[m/s], the time delays are calculated according to Equation (1). Such delays are then programmed into the control unit of the SHMUS so that the guided-waves can constructively interfere in the direction of interest. Additionally, the calculated delays need to accommodate the time precision of the signal generation, which is dependent on the frequency of the generator, $f_{g}=60\;\mathrm{MHz}$ in this case. To this end, the nearest integer number of periods $N_{T_{g,i}}\in N^{0}$ of generation that is introduced in the SHMUS is given by:

(3) $$\begin{array}{c} N_{T_{g,i}}=\left\lfloor \delta_{i}/f_{g}\right\rceil \end{array}$$

where ⌊·⌉ denotes the round-off function to the nearest integer. The excitation signals used in the experiments are generated by a 4 cycle sine tone burst centered at 300kHz with an approximate amplitude of $48\;V_{\mathrm{pp}}$. The SHMUS also allows a comprehensive inspection of a plate-like structure by performing a high number of beamforming tests in order to sweep the area of interest. Here, 37 different paths (i.e., from $0^{{}^{\circ}}$ to $180^{{}^{\circ}}$, every $5^{{}^{\circ}}$) are proposed to be monitored using the 10 PWAS of the phased-array described above in pulse-echo mode, hence emitting and acquiring 37×10=370 ultrasonic signals. This operation takes less than 24 s using PAMELA. Additionally, the calculated delays for such transmission beamforming scenarios are listed in Table 1, where the aforementioned time resolution of the SHMUS can be appreciated.

**Figure 6:**
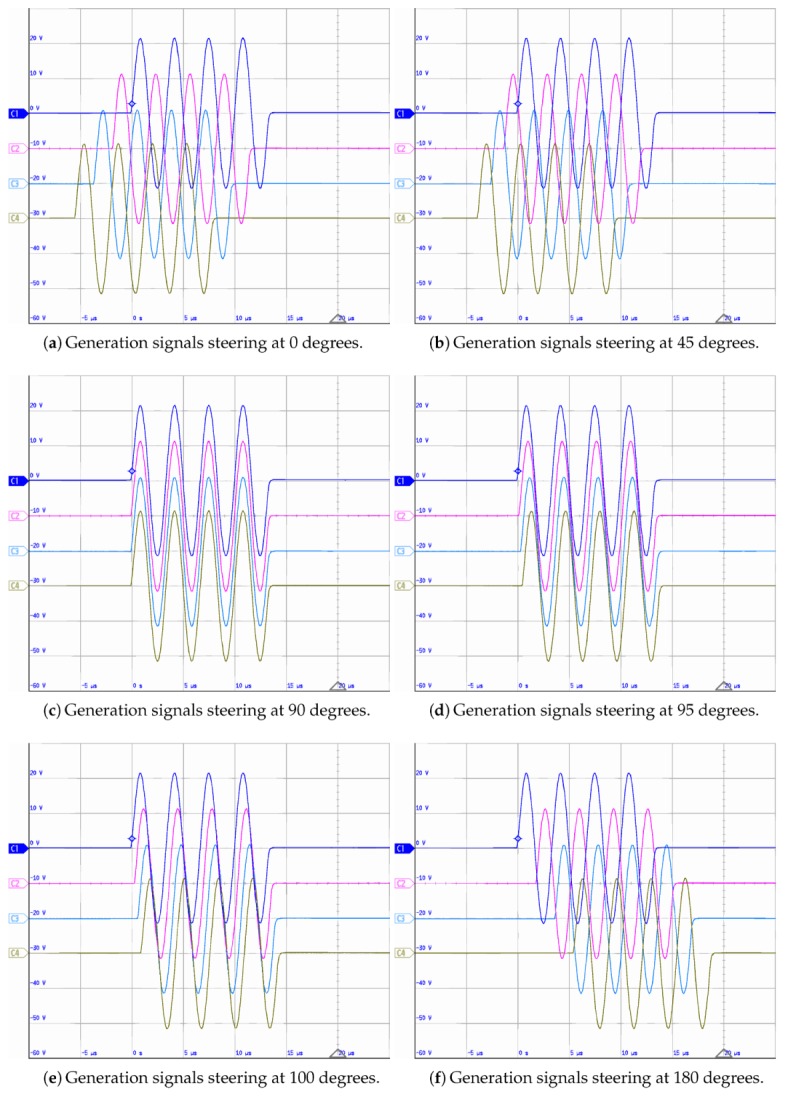
Beamforming excitation signals generated in the structural health monitoring ultrasonic system (SHMUS) and digitized with an oscilloscope. The horizontal axes represent the time with 5 μs/div while the vertical ones show the voltage amplitude with 10 V/div.

Figure 6 shows four of the ten synchronized ultrasonic signals of the SHMUS in order to generate a beam in the following directions: $\alpha =\{0^{{}^{\circ}},45^{{}^{\circ}},90^{{}^{\circ}},95^{{}^{\circ}},100^{{}^{\circ}},180^{{}^{\circ}}\}$ (Figure 6a–f respectively). Note that an oscilloscope with four channels has been used to acquire the excitation signals. These signals are not acquired using SHMUS because it is configured to receive the sensor signal with high resolution, which is of the order of millivolts.

## 4. Experimental Results and Analysis

The experimental assessment of the transmission beamforming includes (1) the effect of the number of actuators, (2) the angular precision and attenuation of the main lobe, (3) the effect of the edges in its inspection, and (4) the analysis of the received signals in the phased-array.

### 4.1. Effect of the Number of Actuators

**Figure 7:**
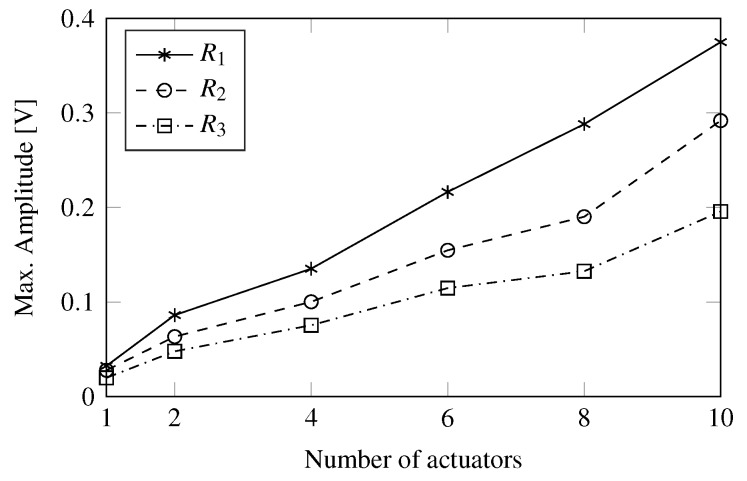
Maximum amplitudes acquired at three different distances from the phased-array (i.e., $R_{1}=170$ mm, $R_{2}=270$ mm, and $R_{3}=400$ mm) in the transmission beamforming tests. The main beam is steered at $60^{{}^{\circ}}$ and different number of actuators are used.

The number of PWAS transducers in the linear phased-array used to generate the beamforming is a key decision variable that is experimentally investigated in the aluminum plate. To this end, the main beam is directed towards $60^{{}^{\circ}}$ using 10, 8, 6, 4, 2, and 1 emitters. In this case, the receiver PWAS are placed at different distances, i.e., C${}_{60}^{R_{1}}$, C${}_{60}^{R_{2}}$, and C${}_{60}^{R_{3}}$. Figure 7 shows the relationship between number of emitter PWAS and the maximum amplitude of the constructive interference created by the transmission beamforming technique. As can be observed, a quasi-linear trend is appreciated for each of the radius, however, the lines are not parallel, and the closer the receiver PWAS is to the phased-array, the higher is the slope of the trend line. This behavior can be explained since the spatial attenuation of the guided-waves when traveling across the structure has a higher influence in the signals than the number of actuators. Note that these measurements were repeated several times, obtaining a negligible variation (less than 1% standard deviation of the acquired amplitude).

Observe also that the amplitudes generated by transmission beamforming are significantly higher than the ones obtained in a single test, i.e., using only one PWAS. This technique allows the guided-waves to travel longer distances until the attenuation results in a low SNR, and hence a greater area of the plate can be explored. Thus, the inspection and monitoring capabilities of the ultrasonic guided-wave based SHM system are supported by this approach, which enhances the SNR level and excites the structure with higher energy. Note also that no saturation point is appreciated in Figure 7 for the maximum amplitude with the number of emitters used in the phased-array. This behavior suggests that, in a scenario where the amplitude of the acquired signal is highly contaminated by background noise, the use of more transducers in the generation of transmission beamforming could provide better quality of the measurements. Theoretically, the use of *n* emitter PWAS, instead of only one, enhance the SNR by 20·log(n) dB. In the previous case, i.e., using 10 PWAS for the generation, the SNR would have been enhanced by 20 dB.

### 4.2. Angular Precision and Attenuation Effects

**Figure 8:**
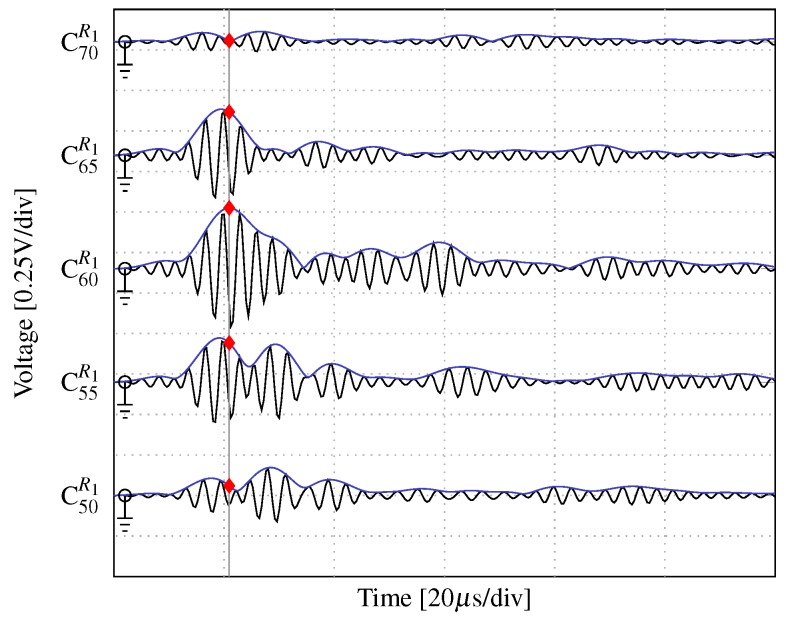
Ultrasonic signals acquired in C${}_{i}^{R_{1}}$ (in black) along with their envelopes (blue curves) and the corresponding time of flight of the measured maximum amplitude (in gray lines and red marks, respectively).

In addition to the number of actuators, the angular precision and attenuation of the main beam are another two key parameters that are experimentally investigated herein. To this end, the SHMUS is configured to steer the main beam towards three different directions, i.e., $i\in \{60^{{}^{\circ}},70^{{}^{\circ}},80^{{}^{\circ}}\}$, and additional PWAS transducers placed at the three different radius, previously described ($R_{1}$, $R_{2}$, and $R_{3}$), are used for the acquisition of the ultrasonic guided-waves. Such receiver transducers are denoted as follows: C${}_{i-10}^{R_{\ell }}$, C${}_{i-5}^{R_{\ell }}$, C${}_{i}^{R_{\ell }}$, C${}_{i+5}^{R_{\ell }}$, and C${}_{i+10}^{R_{\ell }}$, where ℓ∈{1,2,3}. To measure the width of the main beam, the time of flight corresponding to the maximum amplitude at each central sensor (C${}_{i}^{R_{\ell }}$) is measured. Then, the amplitudes of the surrounding positions at the same time of flight are compared, as depicted in Figure 8 for the transmission beamforming test steered at $60^{{}^{\circ}}$ and measured in C${}_{60}^{R_{1}}$. Note that the acquired signals are filtered using a bandpass filter [38] centered at the frequency of excitation (i.e., the resonant frequency of the chosen PWAS) in order to reduce the background noise. The envelopes are obtained by applying the well-known Hilbert transform [39].

**Figure 9:**
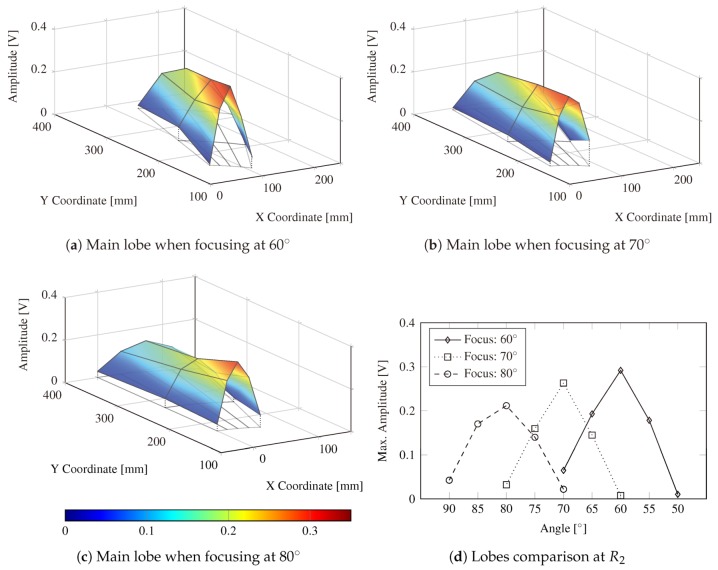
Graphical representation of the main lobes generated by the beamforming technique at three different directions: $60^{{}^{\circ}}$, $70^{{}^{\circ}}$, and $80^{{}^{\circ}}$ in panels (**a**–**c**), respectively. The color bars represent the voltage when the main beam crosses each radius. The PWAS used at the three radius and different angles are represented by the intersections of the radial mesh of the horizontal plane. A comparison of the lobes’ width at $R_{2}$ is shown in panel (**d**).

Figure 9 shows the voltage amplitude surfaces obtained for each beamforming scenario. The results illustrate the angular precision of the main lobe by means of the width of the lobe obtained using 10 PWAS in the phased-array for performing the transmission beamforming. Note that the sensor C${}_{50}^{R_{3}}$ is missing due to the lack of space in the aluminum plate. The width of the main lobe results to be $10^{{}^{\circ}}$ with a significant amplitude (i.e., ≥0.1V, see Figure 9d), although the maximum one is acquired at the steering direction. For instance, the amplitudes registered at radius $R_{1}$ and focusing direction of $60^{{}^{\circ}}$ are still relatively high (i.e., ≥0.2V) at $55^{{}^{\circ}}$ and $65^{{}^{\circ}}$, thus the width of the lobe is assumed to be $10^{{}^{\circ}}$. Analogously, the same behavior is observed at the other two scenarios, i.e., focusing at $70^{{}^{\circ}}$ and $80^{{}^{\circ}}$, and for the three radius. These results highlight the advantage of generating guided-waves with higher amplitude at any desired direction, by which a better information about the structure could be obtained. Additionally, the measured width of the lobe ($10^{{}^{\circ}}$) manifests that, considering 10 PWAS in the phased-array, the monitoring of a structure made of isotropic material using transmission beamforming every $5^{{}^{\circ}}$ could be appropriate for a robust SHM.

Furthermore, it is noticeable that the larger the focusing angle (i.e., closer to the perpendicular line with respect to the phased-array), the lower is the maximum amplitude received at the sensors, as evident from Figure 9d. Thus, when acquiring at C${}_{60}^{R_{1}}$ and focusing at $60^{{}^{\circ}}$ the registered maximum amplitude is higher than the ones obtained in sensors C${}_{70}^{R_{1}}$ and C${}_{80}^{R_{1}}$ when focusing at $70^{{}^{\circ}}$ and $80^{{}^{\circ}}$, respectively. An analogous behavior is identified at further distances (i.e., radius $R_{2}$ and $R_{3}$) from the origin, i.e., the center of the phased-array. This can be explained since the projection of the phased array is wider in angles closer to $90^{{}^{\circ}}$ and narrower when steering towards $0^{{}^{\circ}}$ or $180^{{}^{\circ}}$ direction. Despite the slight loss of signal amplitude, the angular precision is obtained to be the same for every focusing angle.

Based on the results shown in Figure 9, the attenuation effect caused by the wave propagation distance can be also noticed at each beam steering direction. Thus, a loss in amplitude with an increasing distance can be appreciated in the tail of each surface shown in Figure 9. A quasi-linear decreasing trend with the distance is noticed from $R_{1}$ to $R_{3}$ for each of the three focusing directions. Nevertheless, the amplitudes at any radius are significantly higher than the ones obtained when the emission of the ultrasonic signal is carried out by only one PWAS, which result to be between 11.57 and 9.85 times more, as evident from Figure 7. Note that this result may be affected by several factors such as the spacing of the actuators in the phased-array, the type of the PWAS, and the number of PWAS in the emitting phased-array.

### 4.3. Effect of the Edges

The edges of a plate are critical areas for damage onset and propagation. Therefore, an accurate inspection of such area is crucial to ensure a healthy monitoring of the structure in operational service. To investigate the capabilities of the transmission beamforming using the SHMUS in inspecting the edges of the aluminum plate, the amplitudes and width of the main lobe are measured using the lateral sensors denoted by L${}_{k}$ (recall Figure 4a). As an example of such performance, two tests are carried out by steering the main beam towards the position of two receiver PWAS.

**Figure 10:**
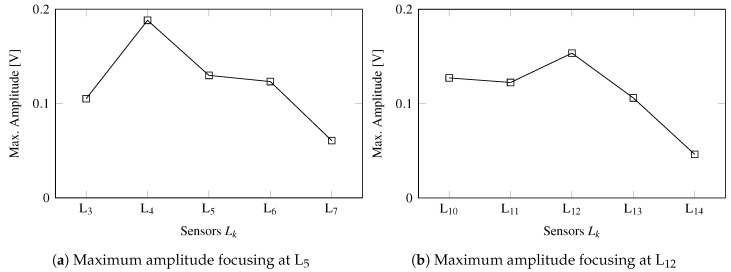
Focusing effect at the edge of the aluminum plate when focusing at sensor L${}_{5}$ in panel (**a**) and at sensor L${}_{12}$ placed in the corner of the aluminum plate in panel (**b**).

First, the system is configured so that the main beam is steered towards L${}_{5}$, placed at the left edge, and the resulting guided-waves are acquired at the neighbor PWAS, i.e., $L_{3},L_{4},L_{5},L_{6},L_{7}$. The maximum amplitudes of the ultrasonic signals are then measured and compared in Figure 10a, whereby a slight deviation in the main beam is identified since the PWAS L${}_{4}$ registers a higher amplitude than L${}_{5}$. This slight deviation is caused by the increased complexity of such area, where the boundary reflections and the irregularities of the edge play an important role in the constructive interference of the transmission beamforming. Nevertheless, the measured amplitudes are significantly high (i.e., ≈0.1V), which still allows a robust SHM of the plate’s edges.

Second, the SHMUS is configured to steer the beam towards the upper left corner, which is the farthest area of the plate from the phased-array. Figure 10b shows the maximum amplitudes measured at L${}_{12}$ and its neighbor receiver PWAS, i.e., $L_{10},L_{11},L_{13},L_{14}$. In this case, the maximum amplitude is measured at L${}_{12}$ (at the corner) with a significant amplitude of 0.153 V, considering that such PWAS is placed 975 mm away from the center of the phased-array.

As is evident from these results, the directionality of the main lobe has not been fully demonstrated when monitoring the entire plate, since the area close to the plate’s edges shows certain limitations. Thus, the beamforming technique should be carefully exploited in the localization of defects in the vicinity of the structure’s boundaries. Notwithstanding this, the enhanced amplitude allows a healthier monitoring since a wider area can be inspected and the features obtained from the signals are less influenced by the background noise, thus potentially reducing the number of false alarms or false positives in the detection of damage.

### 4.4. Acquired Signals

**Figure 11:**
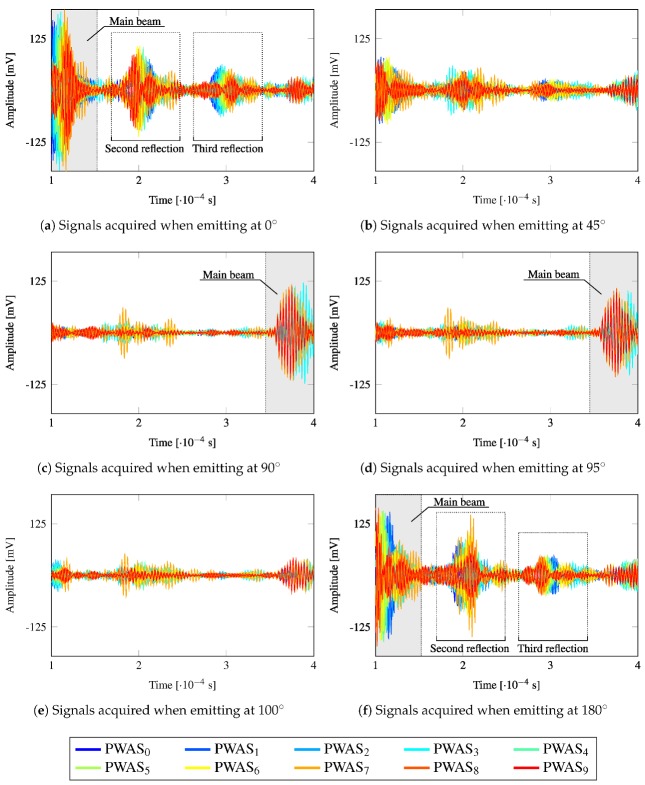
Signals received by the SHMUS in beamforming and pulse-echo mode for different steering directions.

The continuous SHM of the plate is proposed to be performed by transmission beamforming using a linear phased-array of PWAS working in pulse-echo. Therefore, the reception of signals lowly contaminated by noise is crucial for the reliability and accuracy of such monitoring. Figure 11 depicts the measured ultrasonic signals at the phased-array when emitting at $\left\{0^{{}^{\circ}},45^{{}^{\circ}},90^{{}^{\circ}},95^{{}^{\circ}},100^{{}^{\circ}},180^{{}^{\circ}}\right\}$ using the 10 PWAS of the phased-array, according to the generation shown in Figure 6. As is evident from the results, the information obtained in each sensor is different from each other due to the different steering directions and positions in the phased-array. Moreover, the amount of information stemming from a particular beamforming test, devised with an interval of $5^{{}^{\circ}}$, results in 370 different signals, i.e., 37 different directions multiplied by 10 transducers in the phased-array working in pulse-echo. Note that Figure 9 shows an interval of $10^{{}^{\circ}}$, whereas an interval of $5^{{}^{\circ}}$ is proposed to achieve a higher resolution. Given the width of the main beam and that the linear phased-array is placed at the bottom of the plate (see Figure 4), the structure is swept by the beamforming test. Such a high amount of information may also imply a more robust SHM when compared to receiver beamforming technique, e.g., using a round-robin configuration with 10 PWAS transducers, which would lead to 10×10=100 signals with lower amplitude.

Observe that the main beam is clearly received in $0^{{}^{\circ}}$, $90^{{}^{\circ}}$, $95^{{}^{\circ}}$, and $180^{{}^{\circ}}$ directions (Figure 11a,c,d,f, respectively), which corresponds to the first reflection coming from the edges of the plate. Figure 11a,f ($0^{{}^{\circ}}$ and $180^{{}^{\circ}}$, respectively) also show the second and third reflection of the main beam from the lateral edges of the plate. The symmetry shown in the color of the signals (red to blue and blue to red) indicates the origin of the reflection of the main beam, i.e., from the left or right edges. The time of flight of such main beam could be used to estimate the distance to the border of the plate in case the same is unknown. For instance, when the main beam is directed along the Y axis ($90^{{}^{\circ}}$, Figure 11c), the main beam travels a path of (1003−30)×2=1946[mm] (until the plate top edge and its return) at a wave propagation velocity of 5492.26[m/s], i.e., it takes $3.54\times 10^{-4}\;\left[s\right]$. It can be appreciated in Figure 11c that the main beam starts to be received in the phased-array by the previous time of flight. However, the main beam is not received within the chosen time window ($1\times 10^{-4}$ to $4\times 10^{-4}$ [s]) in Figure 11b,e, which correspond to the directions $45^{{}^{\circ}}$ and $100^{{}^{\circ}}$, respectively. In turn, the observed wave packets in these figures that have relatively high amplitude may correspond to the acquisition of secondary lobes. This behavior can be explained since the first rebound takes place from an edge at an angle such that the reflected wave packet does not reach the phased-array back again within such time window. In these cases, the presence of a defect in the first path of the main beam (until the first edge reflection) could be identified in the received signals as a new wave packet. These results highlight the remarkable directionality achieved with transmission beamforming and its superior capability of detecting and localizing damage which has been widely reported in the literature [9,17].

## 5. Conclusions

The capabilities of the transmission beamforming technique in ultrasonic guided-wave based SHM for isotropic materials have been experimentally investigated in this work. To that end, a dedicated hardware has been introduced as SHM system, whereby the ultrasonic signals can be synchronously generated and acquired. Besides, an aluminum plate has been adopted for the empirical investigation of such technique using a phased-array for the generation of ultrasonic guided-waves and several PWAS transducers are used as sensors to acquire the ultrasonic signals, i.e., working in pitch–catch mode.

The use of transmission beamforming has entailed the acquisition of ultrasonic signals with higher amplitude than the experimental procedure using one emitter PWAS. Such amplitudes result to be approximately proportional to the number of emitter PWAS. Therefore, a higher SNR has been obtained. In addition, a remarkable directionality of the main beam has been achieved. These results support the proposed recommendations for SHM consisting of the inspection of a plate using transmission beamforming in pulse-echo mode from $0^{{}^{\circ}}$ to $180^{{}^{\circ}}$ every $5^{{}^{\circ}}$, so 37 tests are carried out. This type of inspection involves a high number of different ultrasonic signals (370 signals if an array of 10 PWAS are used) that are acquired in each transmission beamforming test. Such an amount of signals ensures scanning of the complete structure and supports a reliable and accurate SHM. The investigated capabilities of this technique (i.e., high amplitude, directionality, and amount of information) may also potentially benefit the detection of damage in real-world engineering scenarios where noise is an issue. The performance of the beamforming technique in inspecting the areas close to the plate’s edges has resulted to be more complex. The interpretation of the data that contains multiple boundary reflections could lead to a less accurate damage localization, hence being a limitation of the technique.

Further research work has been devised with regards to: (1) the investigation of the performance of transmission beamforming along with the proposed SHMUS experimental setup in detecting damage in both isotropic and transversely isotropic (e.g., composite) plate-like structures and (2) its ability in monitoring the degradation of structures under accelerated life testing, e.g., fatigue testing, of different materials.

## Figures and Tables

**Table 1:** Time delays and corresponding number of periods of the generator clock for different angles used for the transmission beamforming.

Direction	$0^{{}^{\circ}}$		$45^{{}^{\circ}}$		$90^{{}^{\circ}}$		$95^{{}^{\circ}}$		$100^{{}^{\circ}}$		$180^{{}^{\circ}}$	
Parameters	$\delta_{i}$ [μs]	$N_{T_{g}}$	$\delta_{i}$ [μs]	$N_{T_{g}}$	$\delta_{i}$ [μs]	$N_{T_{g}}$	$\delta_{i}$ [μs]	$N_{T_{g}}$	$\delta_{i}$ [μs]	$N_{T_{g}}$	$\delta_{i}$ [μs]	$N_{T_{g}}$
PWAS${}_{0}$	0	0	0	0	0	0	1.43	86	2.85	170	16.39	984
PWAS${}_{1}$	1.82	110	1.29	78	0	0	1.27	76	2.53	151	14.57	874
PWAS${}_{2}$	3.64	219	2.58	115	0	0	1.11	67	2.21	132	12.75	765
PWAS${}_{3}$	5.46	328	3.86	232	0	0	0.95	57	1.90	113	10.93	656
PWAS${}_{4}$	7.28	437	5.15	309	0	0	0.79	48	1.58	94	9.10	547
PWAS${}_{5}$	9.10	547	6.44	387	0	0	0.63	38	1.26	76	7.28	437
PWAS${}_{6}$	10.93	656	7.73	464	0	0	0.48	29	0.95	57	5.46	328
PWAS${}_{7}$	12.75	765	9.01	541	0	0	0.32	19	0.63	38	3.64	219
PWAS${}_{8}$	14.57	874	10.30	618	0	0	0.16	10	0.32	19	1.82	110
PWAS${}_{9}$	16.39	984	11.59	696	0	0	0	0	0	0	0	0

**Figure A1:**
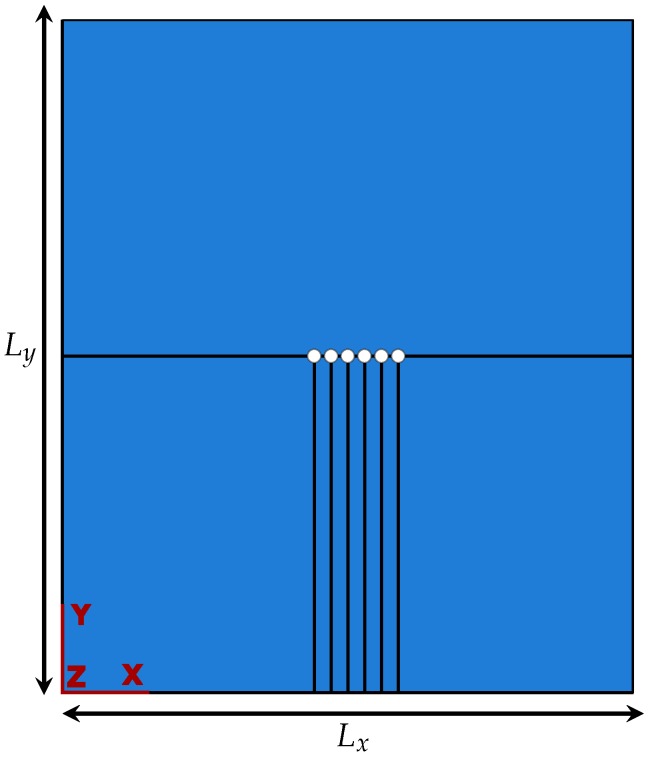
Abaqus model for transmission beamforming simulation.

## Appendix A. Beamforming Simulation Setup

This appendix contains all the necessary information for reproducing the simulation given in Figure 2.

### Appendix A.1. Geometry and Material Properties

The model is a rectangular plate as shown in Figure A1 with the following dimensions:

(A1) $$\begin{array}{c} \begin{array}{c} L_{x}=340\;\mathrm{mm},\;L_{y}=400\;\mathrm{mm},\;L_{z}=1\;\mathrm{mm} \end{array} \end{array}$$

The wave propagates in the x−y plane while *z* is the out of plane direction of the plate. The material is linear elastic isotropic QQ-A-250/5 ‘O’ aluminum with the material properties given in Section 3.2.

### Appendix A.2. Boundary Conditions

A forcing function P(τ) is applied to simulate the six PWAS, which is a four cycle Hanning-windowed sinusoid with the central frequency of excitation $f_{e}=360$ kHz. The forcing function is calculated as follows:

(A2) $$\begin{array}{c} \begin{array}{c} P\left(\tau \right)=w_{H}\left(\sin\left(2\;\pi \;f_{e}\;\tau \right)\right)\;\mathrm{where}\;\tau \in \left[0,\;4/f_{e}\right] \end{array} \end{array}$$

where $w_{H}$ refers to the application of the Hanning window and τ is non-dimensional time. The transient force applied at each transducer is then given as follows:

(A3) $$\begin{array}{c} \begin{array}{c} CF_{i}\left(t\right)=\left\{\begin{array}{cc} 0 & t<\delta_{i}\\ P(\tau ) & t\geq \delta_{i} \end{array}\right. \end{array} \end{array}$$

where i=0,…,5 denotes the elements of the phased-array from left to right and $\delta_{i}$ is the delay for each PWAS for focusing the beam in the positive x− direction. They are calculated using Equation (1). These forces are introduced as tabular amplitudes in Abaqus and then applied as concentrated point forces (CF) in the negative z− direction. The distance between two consecutive PWAS is 10 mm and they are all located in the same plane on the top surface of the plate.

### Appendix A.3. Meshing Parameters

The out of plane point forces will excite both the symmetric (S0) and the antisymmetric (A0) mode. The delays have been calculated for the beamforming of A0 mode. Accurate simulation of A0 mode requires at least five elements through the thickness. The in-plane element edge length (Δl) is calculated from the relation Δl<λ/10 [40], where λ is the wavelength of the slowest mode which is A0 in this case. Here an 8-node general purpose linear brick element (C3D8R) [41], with reduced integration and Δl=0.5 mm, is used. This leads to a total mesh size of 2.72 million elements.

### Appendix A.4. Explicit Step Properties

The dynamic explicit step simulates 200 μs, which is sufficient to observe the main beam forming and reflecting from the boundary as shown in Figure 2. The minimum time step size for a stable FE simulation follows from the fact that a wave must not traverse more than a single element length in one time step [40], hence the condition $\Delta t<\Delta l/V(f_{e})$. Here, $V(f_{e})$ is the propagation velocity of the fastest propagating mode, which is S0 in this case. Therefore, a maximum time increment of 0.01 μs is specified.
